# What changes in Research Ethics in Brazil: Resolution no. 466/12 of the National Health Council

**DOI:** 10.1590/S1679-45082014ED3077

**Published:** 2014

**Affiliations:** * Coordination of Science, Technology and Strategic Inputs, Secretariat of Health of the State of São Paulo, São Paulo, SP, Brazil; Research Ethics Committee of Hospital Ipiranga, São Paulo, SP, Brazil; Research Ethics Committee of Hospital Ipiranga, São Paulo, SP, Brazil

The Resolution no. 196, of October 10, 1996, of the National Health Council (CNS, acronym in Portuguese),^(1)^ approves the regulatory guidelines and norms for research involving human beings and constitutes the first national regulatory mark of ethics applied to research. By means of this resolution, the Brazilian system of ethics review was created, composed by the Research Ethics Committees (CEPs, acronym in Portuguese) and the National Research Ethics Commission (CONEP, acronym in Portuguese), also known as the CEP/CONEP System.

After 15 years, the process of review of CNS Resolution no. 196/96 was initiated. This involved public consultation during the period of September 12 to November 10, 2011, which resulted in 1,890 suggestions sent electronically and the presentation of 18 documents sent by mail. These contributions, duly tabulated, were submitted to the analysis of participants of the extraordinary National Meeting of Research Ethics Committees that produced a document and submitted it to the CNS.

The Plenary of the National Health Council, in its 240th Ordinary Meeting, on December 11-12, 2012, exercising the regulatory powers and rights conferred, revokes CNS Resolutions no. 196/96, no. 303/2000, and no. 404/2008, and replaces by CNS Resolution no. 466, of October 12, 2012,^(2)^ which approves regulatory guidelines and norms to be obeyed as of June 13, 2013 – date of its publication.

The new resolution is divided into 13 parts and is longer and more philosophical, taking into consideration the basic bioethical background, such as recognition and affirmation of dignity, liberty, autonomy, beneficence, non-malfeasance, justice, and equity, among others that seek to guarantee the rights and duties of the research participants, scientific community, and the State.

In the introduction, called “Preamble”, the documents mentioned are the same as those that served as a foundation for Resolution no. 196/96 (the Nuremberg Code, the Declaration of Human Rights, the Declaration of Helsinki of 2000, international agreements on civil and political rights, and international guidelines for biomedical research — Council for International Organizations of Medical Sciences, CIOMS —, besides the Federal Constitution of Brazil. However, new international documents were also incorporated, such as the Universal Declaration on the Human Genome, International Declaration on Human Genetic Data, and the Universal Declaration on Bioethics and Human Rights, although no reference was made to the Declaration of Helsinki in its latest version, of 2008, referring only to the versions from when the use of placebo was not yet flexible (up until 2000).

In part II, “Terms and definitions”, the CNS Resolution no. 466/12 adopts 25 terms, while the previous resolution only cited 16. Some of them are new, such as the “research subject” who is now called “research participant”, in order to designate the individual who, in an informed and voluntary manner, or by explanation to and authorization of his/her legal guardian, accepts being studied. “Research findings” indicates facts or information found by the investigator during the research and that are considered relevant to the participants. “Free and informed assent” is the agreement of the research participant, a child or legally incapable adolescent, free from vices, dependence, subordination, or intimidation. Such participants should be informed as to the nature of the research, its objective, methods, foreseen benefits, potential risks, and the discomfort it may cause, as long as they understand and their singularities are respected. “Integral care” is that care given to treat complications and damage resulting directly or indirectly from the study and “immediate care” is emergency care offered with no burden of any type to the research participant. Another term used is “benefit of the research” which is, by definition, the direct or indirect benefit, whether immediate or posterior, the research participant reaps. The term “sponsor” received a new definition, since in CNS Resolution no. 196/96 it was defined as the individual or legal entity that provides financial support for the research; in the current resolution, it is the individual or private or public legal entity that assists the research by means of financing, infrastructure, human resources, or institutional support. Thus, even in academic studies, the organizations are now seen as sponsors, with all the responsibilities inherent to this title.

In item III, “Ethical aspects of research”, all topics present in Resolution no. 196/96 were maintained, with details added, such as the guarantee to all participants of free access for an unlimited time to the best prophylactic, diagnostic, and therapeutic methods that have proven effective, and the assurance to the women who declare themselves as expressly exempt of any risk of pregnancy, whether due to non-engagement in any sexual practices or engagement in a non-productive manner, the right to participate in research without the mandatory use of contraceptives.

“Informed Consent”, in item IV, had its title altered to “Process of Informed Consent”, that is, an item was incorporated detailing all the steps to be necessarily completed for the person invited to participate in the investigation to be able to manifest him/herself in an autonomous, conscious, free, and informed manner. The document should be drawn up with two copies, with initials on every page, and one of the copies should remain with the person invited. The description of the information process and signing of the Informed Consent Form are mandatory. The requirement for initials already existed, but it was not a part of CNS Resolution no. 196/96.

When analyzing item V, “Risks and benefits”, few modifications were noted and only one inclusion was performed in CNS Resolution no. 466/12. Now the following information has been incorporated: in healthrelated research, as soon as a significant comparative superiority is determined of one intervention over another/others, the investigator should evaluate the need to adjust to or interrupt the study underway, aiming to provide to all the benefits of the better regimen.

In item VI, “On the research protocol”, the new resolution excludes the list of documents that should be submitted to analysis and adds the text:

*The protocol to be submitted to ethical review should only be appreciated if all the documentation requested by the CEP/CONEP System is present, considering the nature and specificities of each research. Plataforma Brasil is the official research launching system for analysis and monitoring of the CEP/CONEP System.*

The list was later published in Operational Norm no. 001/2013,^(3)^ which provides on the organization, function, and procedures for submission, evaluation, and follow-up of the research. In this way, in this item, the *Plataforma Brasil* System is dedicated as an exclusive work instrument.

Item VII, “On the CEP-CONEP System”, did not exist in Resolution CNS no. 196/96. This topic referred to the Research Ethics Committee as “CEP”. The CNS Resolution no. 466/12 defines CEP and CONEP, in addition to emphasizing the character of entirety and partnership of the CEPs/CONEP System, which should act in a cooperative and interrelated fashion.

Item VIII, “On the Research Ethics Committees (CEP)” of the current resolution describes the attributions of the CEPs and includes the following text:

*To evaluate the research protocols involving human beings, with priority on themes of public relevance and strategic interest of the agenda of priorities of SUS, based on epidemiological indicators, providing a duly justified official statement, always guided by, among other things, the principles of impersonality, transparency, reasonability, proportionality, and efficiency, within the timeframes established in an operational norm, avoiding redundancies that delay the analysis.*

The priority of terms with public relevance and of interest to SUS was included, and the timeframes for analysis were removed from the norm and established in Operational Norm no. 001/2013.

We verified that in item IX, “On the National Research Ethics Commission (CONEP)”, its rights are included. The content of CNS Resolution no. 303/2000 was included, which speaks to research on human reproduction. Still in the theme area of research with human genetics, in the previous resolution, whenever genetic material was sent out of the country, it needed to have the official opinion of CONEP. In the present resolution, in cases where there is cooperation with the Brazilian government, the official opinion shall be issued only by the CEP, except when it considers it necessary to forward the issue to the superior agency. Research projects that involve genetically modified organisms, embryo stem cells, and organisms that represent a high collective risk, including the organisms related to them, in the area of experimentation, construction, cultivation, manipulation, storage, release into the environment, and disposal, and protocols on the constitution and function of biobanks for research purposes, will also be the responsibility of the CONEP evaluation. With one alteration in CNS Resolution no. 466/12, in the pertinent item, the text says: “*research with coordination and/or sponsorship originated outside of Brazil, except those cosponsored by the Brazilian Government*”; we understand, then, that any co-sponsorship of the government excludes the need for submission to CONEP. Therefore, projects by students with scholarships from development agencies do not need to be submitted. The process of accreditation of CEPs was excluded from the current resolution; however, it is maintained as a right of CONEP, described in Operational Norm no. 001/2013.

In item X “On the procedure of ethical analysis”, there were only two changes on the competencies of the CEPs: the timeframe for analysis of the project is no longer determined in CNS Resolution no. 466/12, but in Operational Norm no. 001/2013; and under the responsibility of the CEP, filing the documents related to studies for five years is mandatory. In the competencies of CONEP, only one alteration: the non-determination of the timeframe for issuing of the official opinion, which in CNS Resolution no. 196/96 was of 30 days for CEP and 60 days for CONEP. It is important to highlight that the option “approved with recommendations” was removed. CEPs and CONEP should issue official opinions classifying them as “approved”, “pending”, or “rejected”. In practice, since the *Plataforma Brasil* System was implemented, its members were already aware of this change.

In items XI and XII, respectively “Researcher in Charge” and “Other dispositions”, nothing was significantly changed.

The last item, “Specific resolutions and norms”, was added to deal with operational norms that will be published, starting with Operational Norm no. 001/2013, besides the ethical specifications of research in Social Sciences and others, which use their own methodology in these areas, and will be considered in a supplementary resolution due to its particularities.

Within this scenario, it is important to reinforce the idea that CNS Resolution no. 466/12 is not nor could it be a code of rigid rules. It contains guidelines that guide the ethical judgment of the protocols and establish operational norms. Routinely, we have already faced the fact the analysis of ethicality of a research project cannot be dissociated from the analysis of its scientific nature. However, this does not mean that CEP should issue official opinions on the methodology used in the investigation, but rather on the possible ethical implications or repercussions resulting from the methodological options adopted. Beyond this: the dilemmas identified in the protocols and not covered in the guidelines should be the object of reflection and decision of the CEP. This can still count on CONEP's performing its role of supervision, coordination, and orientation of the entire system.

In our opinion, there are some points that should be revised and updated. For example: how can we follow the Declaration of Helsinki without its latest revision? We understand that the revision of this document, published in 2008, revokes the prior revisions. There are other points that should also be elucidated, and therefore, we conclude that Operational Norms should be published to supplement CNS Resolution no. 466/12.

This is because, according to Potter,^(4)^ the best way to deal with dangerous knowledge is to seek more knowledge.
